# Perspectives on climate action and the changing burden of infectious diseases among young Italian doctors and students: a national survey

**DOI:** 10.3389/fpubh.2024.1382505

**Published:** 2024-07-02

**Authors:** Francesco Vladimiro Segala, Francesco Di Gennaro, Lucia A. A. Giannini, Giacomo Stroffolini, Agnese Colpani, Andrea De Vito, Stefano Di Gregorio, Luisa Frallonardo, Giacomo Guido, Roberta Novara, Angela Amendolara, Ilenia Annunziata Ritacco, Francesca Ferrante, Lorenzo Masini, Ilaria Iannetti, Salvatore Mazzeo, Silvia Marello, Nicola Veronese, Federico Gobbi, Roberta Iatta, Annalisa Saracino

**Affiliations:** ^1^Clinic of Infectious Diseases, Department of Precision and Regenerative Medicine and Ionian Area (DiMePRe-J), University of Bari “Aldo Moro”, Bari, Italy; ^2^Doctors4Future, “Chi si Cura di Te?”, Rome, Italy; ^3^Department of Infectious-Tropical Diseases and Microbiology, IRCCS Sacro Cuore Don Calabria Hospital Negrar, Verona, Italy; ^4^Unit of Infectious Diseases, Department of Medicine, Surgery, and Pharmacy, University of Sassari, Sassari, Italy; ^5^PhD School in Biomedical Science, Biomedical Science Department, University of Sassari, Sassari, Italy; ^6^Scientist Rebellion, Verona, Italy; ^7^Geriatric Unit, Department of Medicine, University of Palermo, Palermo, Italy; ^8^Interdisciplinary Department of Medicine, University of Bari, Bari, Italy

**Keywords:** medical education, climate change, planetary health, infectious diseases, eco-anxiety, climate action

## Abstract

**Background:**

The eco-climatic crisis has been defined by the World Health Organization as the “single biggest health threat facing humanity,” influencing both the emergence of zoonoses and the spread of vector-borne and water-borne diseases. The aim of this survey was to explore knowledge, eco-anxiety and attitudes toward the ecological and climate crisis among young Italian doctors and medical students.

**Methods:**

A cross-sectional, multicenter survey was conducted from November 2022 to June 2023, by administering an anonymous questionnaire to Italian doctors and students of medicine. Endpoint of the study was a Knowledge, Attitudes and Practices (KAP) score on ecological and climate crisis (0–20 points). Association between variables and KAP score was assessed by Kruskal-Wallis’ or Spearman’s test, as appropriate, and significant variables were included into ordinal regression model and reported as adjusted odds ratio (aOR) with their 95% confidence intervals (CI).

**Results:**

Both KAP and eco-anxiety scores showed acceptable levels of consistency with Cronbach’s alpha. A total of 605 medical doctors and students living in 19 Italian regions were included in the study. Median age [Q1-Q3] was 27.6 [24.1–31.3] and females were 352 (58.2%). Despite showing good attitudes toward climate action, knowledge gap were found, with 42.5% (*n* = 257) of the respondents not knowing the temperature limits set by the Paris Agreements and 45.5% (*n* = 275) believing that climate change is caused by sunspots. Fears suggestive for eco-anxiety were common. At multivariable ordinal regression, high levels of eco-anxiety (aOR 1.29, *p* = 0.001) and low trust in government action (aOR 1.96, *p* = 0.003) were associated with a higher KAP score. Only one Italian medical school offered an educational module on climate change.

**Conclusion:**

Young Italian doctors and medical students are concerned about the climate crisis but show poor knowledge of these topics. The Italian academic system should urgently respond to this need.

## 1 Introduction

Climate change is a global health emergency. Human activities are leading to a dramatic increase in global terrestrial and marine temperatures resulting in food and water insecurity, sea-level rise and intensification of extreme weather events such as storms, heatwaves, wildfires, drought, floods and cyclones (1). This has the potential to trigger multiple tipping points that would set climate dynamics out of control (2). In addition, uncontrolled urbanization, deforestation and land-use change have caused the disruption of many of the earth’s ecosystems, contributing to drive the ongoing sixth mass extinction event (3, 4) and an upsurge of zoonosis and other infectious diseases (5, 6).

The health impact of the climate crisis is multifactorial and profound. The World Health Organization (WHO) has estimated that, between 2030 and 2050, 250′000 people per year are expected to die as a consequence of climate change-driven malnutrition, malaria, diarrhea and heat alone (7). Also, air pollution is one of the highest risk factors for respiratory and cardiovascular diseases (8), and heatwaves cause every year more morbidity and deaths, especially among the most fragile (9). Extreme climatic events are causing victims both directly and indirectly—due to greater poverty, food insecurity and infectious diseases’ outbreaks—while driving half of world internal displacements in 2022 (10). Mental health disorders related to climate events, such as ecoanxiety and solastalgia, are increasingly common (11). Since human health is deeply affected by both the climate and nature crisis, for the purpose of this work we adopted the umbrella term of “eco-climate crisis.”

Despite a widespread lack of climate-sensitive medical education, clinicians worldwide are already facing the impacts of climate change on human health (12). With a dedicated working group for Planetary Health education, the European Planetary Health Hub has aimed to integrate Planetary Health education at all levels, including the future generation of doctors (13). Several countries, such as Germany, the Netherlands and the United Kingdom, have started to implement Planetary Health courses in their medical school curricula (14–16). In contrast, in other countries such as Italy, an ecologically sensitive medical education is still lacking, likely limiting physicians’ ability to identify and address climate change-related issues in their clinical practice.

As a first step to understand the need for Planetary Health education in the Italian medical school curricula, we performed a survey on the impacts of eco-climatic crisis on human health. Aim of this study is therefore to explore knowledge about the eco-climate crisis, attitudes and practices toward climate action and feelings of eco-anxiety among young Italian doctors and medical students.

## 2 Materials and methods

### 2.1 Study design and population

We conducted a cross-sectional, multicenter survey from November 2022 to June 2023, by administering an anonymous structured questionnaire. Target population for the survey were Italian medical students, medical residents and doctors. Different typologies of postgraduate medical specialties were classified into clinical, non-clinical and surgical areas as for the Italian Ministry of University and Research classification (DM 68/2015). Italian regions were categorized into Northern, Central, and Southern Italy following the classification system utilized by the Italian National Institute of Statistics (17).

Study population was recruited by convenience sampling. To minimize selection bias, we adopted a multicenter design with a broad geographical setting (19 regions) and we conducted a multivariate analysis accounting for potential confounders. Given the descriptive purposes of the study, no *a-priori* sample size estimation was conducted. Written informed consent was obtained prior to survey administration. To understand to which extent Italian medical schools were addressing the issue of climate crisis impact on human health, publicly available curricula of all Italian Medical Universities were reviewed in November 2022 to identify modules related to climate change. The web-based survey was administered through Redcap (18) and, to prevent multiple submissions, all respondents were advised to complete the survey only once.

Ethical approval was granted by the Research Ethics Committee “Azienda Ospedaliero-Universitaria Consorziale Policlinico,” protocol number “009129–24/04/2023.”

### 2.2 Questionnaire development

Development of the questionnaire was informed by a literature review. Overall, the questionnaire comprised a total of 41 questions, 20 of them used for constructing the Knowledge, Attitudes and Practices (KAP) score. A KAP design was chosen to promote comparability of the results and to facilitate result interpretation and dissemination. The Knowledge section focused on common climate change misconceptions (19), major international agreements and policies, and basic notions about the link between the eco-climate crisis, human health and infectious diseases. One point was awarded for each correct answer or for responses demonstrating a positive attitude or practice toward climate action, with a maximum achievable score of 20.

The questionnaire also featured a section on eco-anxiety, comprising seven items structured as Likert-style questions. This section delved into the participants’ concerns regarding their own or their families’ direct involvement in major catastrophic events linked to climate change. Each question comprised 5 graded answers, to which a score ranging from 0 (“not concerned”) to 4 (“extremely worried”) was assigned. As a result, eco-anxiety was quantified with a score ranging from a minimum of 0 to a maximum of 35 points.

Additional items focused on demographics (4 items) occupation-related information (8 items) and zoonosis and spillover risk (2 items). Full question list is provided in the Supplementary Tables 1, 2.

### 2.3 Statistical analysis

A descriptive analysis was performed to define the distribution of demographics and occupation-related characteristics of the sample. Different typologies of postgraduate medical specialties were classified into clinical, non-clinical and surgical areas as for the Italian Ministry of University and Research classification (DM 68/2015). For numerical variables, normality was assessed with the Shapiro–Wilk test. Missing data points were identified and documented during the descriptive analysis phase.

KAP score was used as the dependent variable. Spearman-rank test was used to compare groups for continuous variables, while a Kruskal-Wallis test was applied for categorical variables. An ordinal regression model was implemented as follows. KAP score was considered as a dependent variable and each one of the available factors at the baseline evaluation as independent variables (univariate analysis). In the multivariate analysis factors with a *p*-value <0.05 by univariate analysis were included. Multicollinearity among covariates was assessed through the variance inflation factor, taking a value of 2 as cut-off to exclude a covariate. However, no variable was excluded according to this pre-specified criterion, suggesting that missing data did not significantly affect the multicollinearity of the model. Odds ratios (ORs) as adjusted odds ratios (adj-ORs) with 95% confidence intervals (CIs) were used to measure the strength of the association between factors at the baseline and KAP score. All statistical tests were two-tailed and statistical significance was assumed for a *p*-value <0.05. Statistical analyses were performed using R Statistical Software (v4.1.3; R Core Team 2021) in R Studio Version (20).

## 3 Results

### 3.1 Demographics

Out of a total of 613 individuals who were invited to complete the questionnaire, 605 provided their consent to participate in the study. The study population encompassed 264 medical students, 194 residents, and 108 specialists. The majority of the medical residents and specialists were employed in the clinical area (*n* = 206, 60.4%), followed by area of clinical services (*n* = 49) and surgical area (*n* = 33). The average age was 27.6 years (interquartile range, Q1-Q3, 24.1–31.3), and 58.2% (*n* = 352) of the participants identified as female. The participants were spread across different regions of Italy, including 100 (16.5%) from Central Italy, 174 (28.8%) from Northern Italy, and 314 (51.9%) from Southern Italy.

### 3.2 Knowledge

Two-hundred fifty seven participants (42.5%) were not able to identify the limits to global temperature increase set by the Paris Agreement (21), and 59.2% (*n* = 358) did not agree that canceling the debt of poorer countries could constitute a valid climate justice policy. Only 16.4% (*n* = 99) could identify influenza as a zoonosis, despite 71.2% (*n* = 431) correctly recognized deforestation as a driver for spillover. Misconceptions about climate change were common: 45.5% of the sample did not express disagreement with the statement “climate change is caused by sunspots” and 15.4% (*n* = 93) either agreed (5.9%, *n* = 36), strongly agreed (2.5%, *n* = 15), or could not take a position (6.45%, *n* = 39) regarding the sentence “The climate change is part of a natural cycle that has always existed, where human activities play only a marginal role.” Detailed responses to all questionnaire items, stratified by occupation, can be found in the Supplementary material. Curricula of all Italian medical universities available online were reviewed on September 15th, 2023. We found only one institution (University of Turin) offering a course explicitly targeting the impacts of climate change on human health.

### 3.3 Attitudes and practices

**Figure 1:**
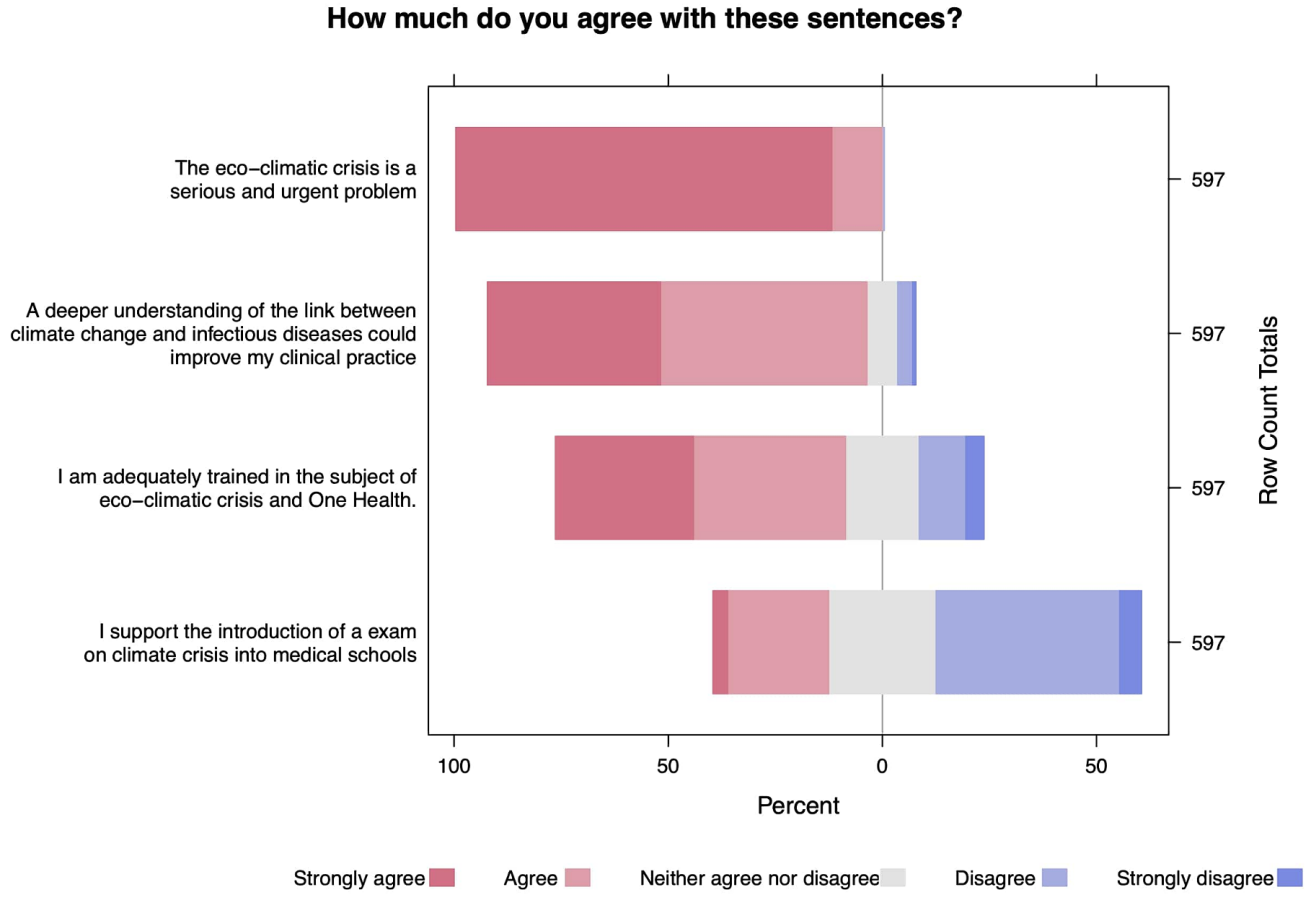
Likert-style responses to selected questions.

**Figure 2:**
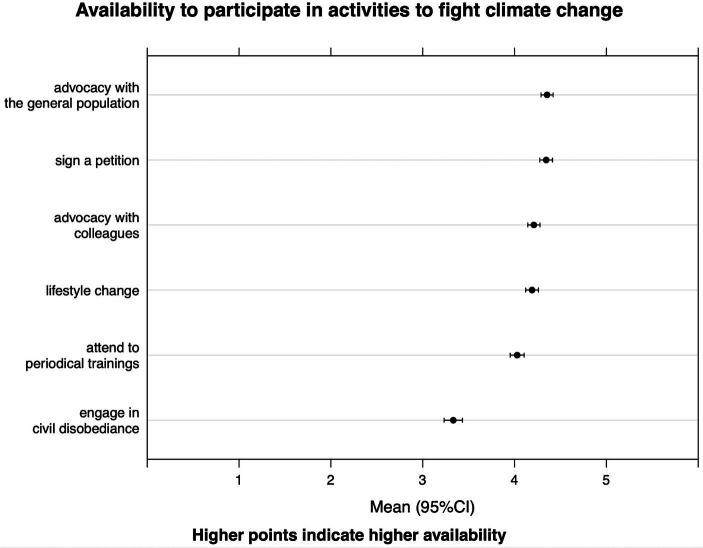
Willingness to engage in selected activities in support for the climate. Each dot represents the mean of the responses from 1 (“minimum availability”) to 5 (“maximum availability”). Tick lines show 95% confidence intervals.

Almost all of our population (*n* = 601, 99.3%) considered the climate crisis an important and urgent problem (Figure 1) and did not agree on the fact that governments are taking adequate action to address it (*n* = 535, 88.43%). Despite this, only 79 (11.6%) people thought that there is nothing left to do to limit the consequences of the eco-climate crisis. In terms of practices (Figure 2), the majority of the population declared to be “available” or “very willing” to adopt carbon-saving lifestyle choices (*n* = 535, 88.4%) and to participate in advocacy activities among coworkers (*n* = 540, 89.3%) and the general population (*n* = 559; 92.4%), while they were generally less prone to engage in civil disobedience (*n* = 279, 46.1%) and to introduce an exam dedicated to climate change and health into medical curricula (*n* = 406, 67.1%).

### 3.4 Eco-anxiety

The fear that the eco-climate crisis would impact the personal life of the respondents and/or of their family member was high, with a median overall eco-anxiety score of 26/35 (Q1-Q3, 22.3–28.0). In particular, 530 (87.6%) participants declared to be concerned (38.3% “worried” and 49.3% “extremely worried”) that human activities will lead to new pandemics in the near future, and 79.5% (32.9% “worried” and 32.9% “extremely worried”) were afraid that vector-borne diseases such as malaria or dengue will become endemic in Italy. Also, 83.9% (39.2% “worried” and 44.7% “extremely worried”) manifested concern that climate change will lead to political instability and wars. Almost the entirety of our population (94.4%, *n* = 571) declared to be afraid (32.4% “worried” and 62% “extremely worried”) that the eco-climate crisis will affect the health of present and future generations. Cronbach’s alpha for eco-anxiety score was 0.84 (95%CI 0.82–0.86), indicating high level of internal consistency.

### 3.5 Bivariate and multivariate analysis for high KAP score

As shown in Table 1, the variables that resulted associated with higher KAP score at bivariate analysis were being female (*p* = 0.012), demonstrating higher eco-anxiety, and lower trust in government action to mitigate climate change. Multivariable ordinal regression analysis confirmed the association with low trust in government action (aOR 0.51, 95%CI 0.32–0.79) and higher eco-anxiety (aOR 1.29, 95%CI 1.24–1.35). The overall Cronbach’s alpha for the KAP score, calculated for a data set of 605 respondents, was 0.71 (95%CE 0.67–0.74), indicating an acceptable level of internal consistency.

**Table 1:** Descriptive statistics, bivariate analysis and ordinal logistic regression for higher KAP score.

	Overall (*N* = 605)	KAP score, Median (Q1-Q3)	*p*-value for higher KAP score^a^	Ordinal regression analysis aOR (95% CI), *p*-value
Gender				
Female	352 (58.2%)	15 (13–16)	**0.012**	Ref
Male	246 (40.7%)	14.5 (12–16)		0.831 (0.62–1.11) *p* = 0.215
Non-binary	4 (0.7%)	17 (16.5–17)		2 (0.32–12.15) *p* = 0.443
Age				
Median [Q1, Q3]	27.6 [24.1, 31.3]	–	0.149	–
Area				
Central Italy	100 (16.5%)	15 (13–16)	0.357	–
Northern Italy	174 (28.8%)	15 (13–16)		–
Southern Italy	314 (51.9%)	15 (12.25–16)		–
Missing	17 (2.8%)	–		–
Position				
Student	264 (43.6%)	15 (12–16)	0.518	–
Medical resident	194 (32.1%)	15 (13–16)		–
Medical specialist	108 (17.9%)	15 (12–17)		–
Other	39 (6.4%)	15 (13–16.5)		–
Medical area (*n* = 341)				
Clinical area	206 (60.4%)	15 (13–16)	0.14	–
Clinical services area	49 (14.4%)	14 (11–16)		–
Surgical area	33 (9.7%)	13 (12–16)		–
Missing	53 (15.5%)	-		–
Eco-anxiety score				
Median [Q1, Q3]	20.0 [17.0, 22.0]	**-**	**<0.001**	1.29 (1.24–1.35), ***p* < 0.001**
Trust in government action to mitigate climate change				
No	535 (88.4%)	15 (13–16)	**<0.001**	Ref.
Yes	70 (11.6%)	13 (11–15)		0.51 (0.32–0.79), **p = 0.003**
Belief that nothing can be done to contrast the climate crisis				
No	574 (94.9%)	15 (13–16)	0.616	–
Yes	31 (5.1%)	15 (12–16)		–

^a^Spearman test and Kruskal-Wallis test, as appropriate. Statistical significant results (*p*> 0.05) are highlighted in bold.

## 4 Discussion

In this study, Italian students of medicine and young doctors showed a positive attitude toward climate change, despite a demonstrating a substantial gaps in knowledge about its causes and consequences on human health. Four respondents out of five were not able to identify the 1.5°C threshold included in the Paris Agreement. Misconceptions about climate-change causes were widespread, including almost half of the respondents attributing a causative role to sunspots and an alarming one in six participants who believed that human activities played only a marginal role in shaping the current climate scenario. Also, only six in 10 persons being able to identify intensive farming as a risk factor for spillover, and a stunning 84% of the sample not being able to recognize influenza as a zoonotic disease. Also, despite the recent scientific debate fostered by the pandemic (22), the majority of the population did not classify COVID-19 as a disease with zoonotic origins (23, 24). To the best of our knowledge, this study represents the first attempt to assess the awareness of physicians and medical students regarding the influence of human-induced ecological degradation on the susceptibility to infectious diseases, which represent roughly 75% of all newly emerging infectious diseases described in the last four decades (25).

The poor knowledge reported in our study is consistent with other surveys conducted among Italian high school students (26), and with a multinational survey conducted on the general population (27). Moreover, our rate of respondents neglecting the role played by human activities is almost identical to the one reported in a large multinational survey conducted on 4,654 health professionals in 2020 (28). Compared to this study, however, our sample exhibited a higher level of engagement (99.3% vs. 75%), with almost all respondents acknowledging the urgency to address the impact of the eco-climate crisis on human health.

In this study, the concern that the eco-climate crisis will have an increasingly negative impact on participant’s health was widespread. Italy is highly vulnerable to climate change, and extreme weather events—such as the 2023 Northern Italy drought, Emilia-Romagna floods, summer wildfires and heathwaves (29). Also, incidence of vector-borne illnesses like Lyme disease (30) and dengue fever (31) is increasing, and clinicians are already witnessing rising heat-related mental health risk (32). All this is fostering a general rise in climate-related fear and anxiety (33).

A peculiar finding of our study is a strong correlation between eco-anxiety with higher eco-climate awareness and positive attitude toward eco-climate crisis. This finding is in line with a multinational survey exploring the correlation between climate-sensitive mental health issues and pro-environmental action (34). Also, similar surveys conducted on other key climate change stakeholders, such as government officers, private sector employees, and NGO operators, showed similar results in terms of climate action engagement.

Physicians occupy a unique position in society, providing them with a profound opportunity for climate action (12). Engagement of the medical community into advocacy and climate activism might accelerate system change, considering their social respectability and their individual experience as daily witness of the effects of climate change on human health (35). This can be achieved on both the individual and societal level, by prescribing low-carbon lifestyle choices and advocating for broader political engagement. To achieve this, physicians should have basic knowledge of the key drivers of human-induced climate change, as well as a robust understanding of how increasingly warmer global temperatures and biodiversity loss impact various aspects of human health. These aspects include, but are not limited to, heat stress risk, mental health, food security, and the suitability for water-borne and vector-borne diseases, and pandemic risks (36).

Our study suggests that Italian doctors are sensitive to the threat posed by the eco-climate crisis and are prone to be engaged into climate action. However, they still lack specific education. Medical institutions and scientific societies are recognizing that it is impossible to separate human health from the health of other living beings and the entire planet. Our results emphasize that is essential to adopt a more comprehensive approach to health such as that the one conceptualized by Planetary Health (37), which leads us to seek the highest standard of health and equity by taking into account both human systems (such as the political, economic and social ones) and the natural systems of the planet. The findings and methodology of this study can be applied to other countries by serving as a model for assessing the knowledge, attitudes, and practices regarding climate change and its health impacts among medical professionals. The survey approach, including the KAP score and eco-anxiety assessment, can be adapted to different cultural and educational contexts to identify gaps in climate-related medical knowledge and the emotional responses of medical students and professionals. This can inform targeted policy changes in medical curricula to better equip future physicians globally to address climate-related health issues, potentially involving them actively in mitigation strategies. In this context, the COVID-19 pandemic contributed to raise awareness among healthcare personnel about the impact of ecological degradation on human health, while presenting an unprecedented opportunity to accelerate global climate mitigation efforts (38).

The limitations of this study are notable and should be carefully considered when interpreting the results. The use of convenience sampling may lead to selection bias, as it might not accurately reflect the broader population of Italian medical professionals and medical students. To minimize selection bias, we implemented a multicenter design encompassing 19 Italian regions and conducted a multivariate analysis to control for potential confounding factors. This method also potentially limits the generalizability of the findings to other medical communities or geographical regions, even with the relatively high numerosity achieved by the study. Also, the reliance on self-reported data can introduce response bias, where participants might provide answers they perceive as socially acceptable rather than those that reflect their true behaviors and beliefs. Furthermore, the cross-sectional design of the study captures attitudes and knowledge at a single point in time, which means it cannot account for changes over time or establish any causal relationships. Finally, while the questionnaire was informed by a literature review, the possibility of overlooking key aspects of climate change knowledge or attitudes that are relevant to medical professionals cannot be discounted. However, this limitation was counterbalanced by a good internal consistency of the outcome score.

## 5 Conclusion

In our study, despite showing poor knowledge of Planetary Health and climate change drivers, Italian physicians and medical students demonstrated interest and considered the issue important. Furthermore, they showed a generally positive attitude and willingness to engage in pro-environmental activities. Policymakers should adopt a new approach to medical education that equips doctors with the ability to recognize climate and ecological risks and integrate principles of planetary health into their clinical practice. The medical education system should empower our physicians to define the boundaries within which humanity can thrive.

## Data availability statement

The raw data supporting the conclusions of this article will be made available by the authors, without undue reservation.

## Ethics statement

The studies involving humans were approved by “Azienda Ospedaliero-Universitaria Consorziale Policlinico,” protocol number “009129–24/04/2023.” The studies were conducted in accordance with the local legislation and institutional requirements. The participants provided their written informed consent to participate in this study.

## Author contributions

FS: Conceptualization, Data curation, Formal analysis, Methodology, Supervision, Writing – original draft, Writing – review & editing. FrG: Conceptualization, Supervision, Validation, Writing – review & editing. LG: Supervision, Writing – original draft, Writing – review & editing. GS: Conceptualization, Data curation, Writing – review & editing, Methodology, Supervision. AC: Data curation, Writing – review & editing. AV: Data curation, Methodology, Writing – review & editing. SG: Data curation, Writing – review & editing. LF: Data curation, Writing – review & editing. GG: Data curation, Writing – review & editing. RN: Data curation, Writing – review & editing. AA: Data curation, Writing – review & editing. IR: Data curation, Writing – review & editing. FF: Data curation, Writing – review & editing. LM: Data curation, Investigation, Methodology, Writing – review & editing. II: Data curation, Investigation, Writing – review & editing. SaM: Data curation, Investigation, Writing – review & editing. SiM: Data curation, Writing – review & editing. NV: Data curation, Supervision, Validation, Writing – original draft, Writing – review & editing. FeG: Supervision, Visualization, Writing – review & editing. RI: Supervision, Validation, Writing – review & editing. AS: Methodology, Resources, Supervision, Validation, Visualization, Writing – review & editing.
